# Optimizing the Cas13 antiviral train: cargo and delivery

**DOI:** 10.15252/emmm.202217146

**Published:** 2023-05-25

**Authors:** Shruti Sharma, Cameron Myhrvold

**Affiliations:** ^1^ Department of Electrical and Computer Engineering Princeton University Princeton NJ USA; ^2^ Department of Molecular Biology Princeton University Princeton NJ USA

**Keywords:** Microbiology, Virology & Host Pathogen Interaction

## Abstract

The severe acute respiratory syndrome coronavirus 2 (SARS‐CoV‐2) pandemic in 2020 highlighted the need for rapid, widespread responses against infectious disease. One such innovation uses CRISPR‐Cas13 technology to directly target and cleave viral RNA, thereby inhibiting replication. Due to their programmability, Cas13‐based antiviral therapies can be rapidly deployed to target emerging viruses, in comparison with traditional therapeutic development that takes at least 12–18 months, and often many years. Moreover, similar to the programmability of mRNA vaccines, Cas13 antivirals can be developed to target mutations as the virus evolves.

## Graphical abstract

This Commentary discusses the development and potential clinical translation of the Cas13‐based antiviral therapies.

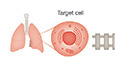

The success of Cas13‐based antiviral therapies depends on two hand‐in‐hand points, the cargo and delivery (Fig 1):

1. The choice of Cas13 ortholog is critical to ensure efficacy.
2. Delivery of Cas13 to relevant tissues is rate‐limiting.

**Figure 1:**
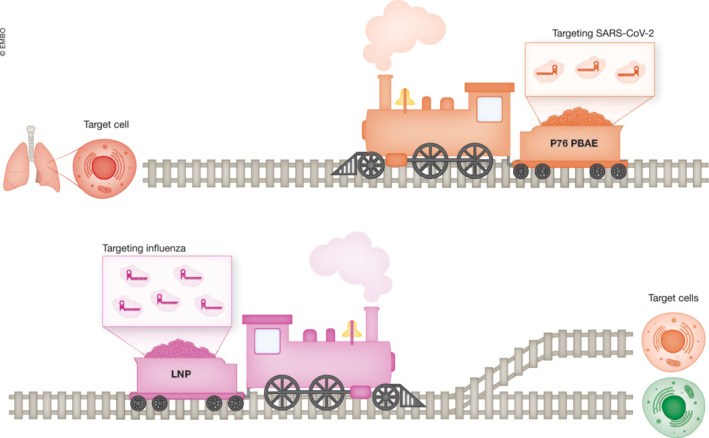
Overview of Cas13‐based antiviral therapies RNA antiviral therapies consist of a crRNA that targets the virus encapsulated within a polymer, which can be inhaled to fight a viral infection. To represent the hand‐in‐hand process of cargo and delivery for antiviral therapy development, we create a metaphor using locomotive trains to depict the process. Each train is targeting a virus with a specific Cas13 RNA ortholog. There are three groups of Cas13 orthologs used for antiviral therapies: Cas13a, Cas13b, and Cas13d. Cas13a is the largest Cas13 ortholog, Cas13b is smaller than Cas13a, and Cas13d is the smallest. Cas13b has a unique 3′ to 5′ CRISPR RNA orientation, in contrast to Cas13a and Cas13d. Depending on the functionality needed, different orthologs have been tested for antiviral therapies. The orthologs are encapsulated in a polymer coating for delivery, such as the poly‐β‐amino‐thio‐ester polymer (P76 PBAE, top), which was shown to be effective against SARS‐CoV‐2 when nebulized into the lungs of mammals. Alternatively, a lipid nanoparticle (LNP, bottom) can be used for encapsulation. The train carries the necessary Cas13 orthologs and CRISPR RNAs (cargo), enabling delivery to target cells. For targeting SARS‐CoV‐2, particles are nebulized to allow for delivery to target cells in the lung. Multiple cell types can be targeted with the same cargo.

There has been progress in both these areas, and research on the most effective choice of Cas13 orthologs predates the pandemic. In a study by the Sabeti group, hundreds of crRNAs were evaluated to determine Cas13s antiviral activity (Freije *et al*, 2019). Their findings catalyzed research in the design of Cas13 viral strategies in time for the pandemic. In response to SARS‐CoV‐2, the Trapani group studied the impact of the PspCas13b ortholog to silence the virus (Fareh *et al*, 2021). The PspCas13b ortholog was chosen for its long spacer sequence, and the group saw the ortholog's relative tolerance to mutations in the target sequence. The spike mRNA was targeted by the crRNA, and high silencing efficiency was achieved with reprogramed CRISPR‐PspCas13b to suppress ancestral variants in human and monkey epithelial cells (Fareh *et al*, 2021). Also in response to the pandemic, the Qi group designed an antiviral “PAC‐MAN” strategy with Cas13d orthologs and used genome‐wide screening to target conserved regions in the viral sequence (Abbott *et al*, 2020). Cas13d was chosen instead of other orthologs and proteins due to its small size and high catalytic activity. However, key limitations for *in vivo* delivery exist for Cas13 antiviral strategies as they have for Cas9 and gene therapies more broadly, as we discuss below.

The next crucial part is the administration and delivery of Cas13‐based antiviral therapies. In research by the Santangelo group, it was shown that antiviral therapies can be delivered directly to lungs in rodents through a minimally toxic polymeric coating carrying its RNA cargo. Over a hundred polymeric nanoparticle formulations were developed to deliver the RNA therapies to the lungs, with a poly‐β‐amino‐thio‐ester polymer exhibiting efficacious results. This work highlights the promising use of polymeric coatings to deliver antiviral therapies (Rotolo *et al*, 2022). Blanchard *et al* also employed polymeric formulations to target influenza A and SARS‐CoV‐2 infections in mice and hamsters. The study found success using LbuCas13a mRNA to mitigate respiratory infections with both influenza A virus and SARS‐CoV‐2 (Blanchard *et al*, 2021). While there is promise, these studies have yet to show a complete cure of infection. Combining research on optimal strategies on the design of crRNAs Cas13 orthologs and delivery will allow for further progress to combat infectious disease.

CRISPR‐Cas13 antivirals have yet to be administered in humans; yet, there has been significant progress with CRISPR‐Cas9 in recent clinical trials (Gilmore *et al*, 2021). CRISPR‐Cas9 treatments were administered in humans for treatment of transthyretin amyloidosis (Gilmore *et al*, 2021). Cas9 and Cas13 are both programmable nucleases, though Cas13 targets and cuts RNA instead of DNA. In this way, Cas13 should be a “safer” nuclease as it targets nonheritable RNA instead of genetic material. Cas13's high specificity, programmability against mutations, and lack of off‐targets make it a promising tool for clinical translation in the fight against infectious diseases.

## Author contributions

**Shruti Sharma:** Writing – original draft; writing – review and editing. **Cameron Myhrvold:** Funding acquisition; writing – original draft; writing – review and editing.

## Disclosure and competing interests statement

C.M. is a cofounder of Carver Biosciences, a startup company developing Cas13‐based antivirals, and holds equity in Carver Biosciences. The remaining authors declare no conflict of interest.
